# Conditions for adherence to videoconference-based programs promoting adapted physical activity in cancer patients: a realist evaluation

**DOI:** 10.1186/s13012-024-01338-y

**Published:** 2024-01-29

**Authors:** Olivier Aromatario, Linda Cambon, François Alla, Alexis Imbert, Camille Pouchepadass, Nathalie Renvoisé, Sarah Dauchy, Cécile Charles

**Affiliations:** 1 INSERM, Population Health Research Center (BPH), U1219, Mérisp/PHARES, Team Labelled Ligue Contre le Cancer, CIC1401, University of Bordeaux, F-33000 Bordeaux, France; 2 Gustave Roussy Cancer Center, Paris, France; 3 University Hospital of Bordeaux, F-33000 Bordeaux, France

**Keywords:** Physical activity, Videoconference, Oncology, Health promotion, Realist evaluation

## Abstract

**Background:**

Although moderate physical activity (PA) during cancer treatment has been associated with improved quality of life, reduced side effects, and even lower mortality, many barriers to successful implementation remain. Digital technology has been perceived as an effective lever for overcoming access and motivational issues but few studies have been performed to confirm this assumption. The “VISIO-AJUST” study explored the factors affecting the conditions of adherence to video-conference-based PA programs in patients undergoing cancer treatment.

**Methods:**

The VISIO-AJUST study was based on a qualitative successive case approach, guided by the principles of the realist evaluation, and applied to two French programs of PA, following three main steps: (1) Identification of factors likely to influence conditions of patients’ adherence; (2) Elaboration and testing of explanatory “Context-Mechanism-Outcome (CMO)” configurations; (3) Refinement of CMO configurations, in order to understand what, how, for whom, and under what circumstances video-conference-based PA programs work.

**Results:**

Five main CMO configurations were found to be associated with adherence to video-conferencing-based PA programs, promoting (i) accessibility and a supportive environment for adapted physical activity, (ii) a setting conducive to sociability despite distance, (iii) Confidence and security of practice, (iv) a combination of several motivational levers in favor of continuity of effort and progress, (v) regularity of the sessions, progressiveness in the effort and evaluation of progress as a basis for the adoption of a regular physical practice.

**Conclusion:**

This study provides original insights into the use of videoconferencing to enable patients to improve their PA during cancer treatment. Future research with long-term follow-up would allow for a better understanding of the key conditions promoting sustainable behavior change.

**Supplementary Information:**

The online version contains supplementary material available at 10.1186/s13012-024-01338-y.

Contributions to the literature

- Moderate physical activity during cancer has been linked to many positive effects but there are many barriers to being physically active during or after treatment
- Remote video conferencing is rapidly developing as a means to increase physical activity. Data on their implementation are lacking.
- Theory-based evaluations identify key implementation modalities for complex interventions
- The study of the implementation of physical activity promotion programs via videoconferencing during cancer treatment clarifies the conditions for patient adherence
- The study shows 5 key elements of patient adherence conditions in physical activity development programs via videoconferencing

## Background

In oncology, regular physical activity of moderate intensity during treatments has been associated with a decrease in the severity of side effects, maintenance of good physical condition and capacities of autonomy, as well as an improvement of quality of life and anxiety-depressive symptoms [1]. An association between physical activity and reduction of mortality has also been shown in breast, colorectal, and prostate cancers [2]. However, cancer-related symptoms and treatment side effects remain substantial barriers to physical activity. In particular, fatigue and pain can affect up to 80% and 50% of patients, respectively, during and beyond treatment [3, 4]. In this context, common psychological barriers to physical activity may be strengthened, such as a lack of self-confidence and/or motivation, and negative representations towards physical activity. Barriers can also be related to environmental and organizational conditions (i.e., climatic and temporal constraints, distances and transport, disparities in terms of access to appropriate structures, financial resources, and social support) [5]. Moreover, recommendations from oncologists about the benefits of physical activity have been associated with an increase in the level of physical activity in disease-free breast and prostate cancer patients. However, a number of obstacles exist preventing the integration of this counseling activity into routine clinical care, some of which are linked to practitioners themselves, including a lack of time, having the impression of not being sufficiently trained in this type of communication, lack of knowledge about official recommendations, or doubts about the effectiveness of counseling on behavioral change [6–9]. The results of Park and colleagues’ study suggested that advice alone was insufficient for promoting physical activity and should be combined with an intervention to be efficient [10]. Moreover, Marthick and colleagues’ study [11] highlighted the positive impact of regular interactions between patients and health professionals on the improvement of patient engagement in digital health interventions (personalized messages perceived as helpful and motivational), and the willingness of some patients to supply their activity data to health professionals for individualized feedback. The importance of the medical professional in promoting physical activity is clear; the French National Institute of Health and Medical Research [12] stressed in its report on physical activity and chronic diseases the importance of the influence of the *"medical professional”*, but also of *“peers and family on motivation and effective physical activity practice”* for cancer population.

For these reasons, we hypothesized that the interactions between cancer patients and their oncologist, their peers, and their relatives would be key contextual factors, with a particularly important influence on the mechanisms triggered by the programs under study. In addition, in recent years, the use of digital technologies (Internet, mobile applications, e-health tools, etc.) has played a part in the evolution of relations between patients, their families, and caregivers. This technology has been considered a promising lever that can be activated to overcome access issues to supportive care [13, 14]. Despite a rise in the development and use of video-conferencing technologies during the COVID-19 pandemic, there is a lack of data available on the extent to which these digital devices improve adherence to adapted physical activity in patients with cancer in real-life conditions.

Our approach operates under the assumption that it is not just the technology or program which affects adherence to physical activity, but rather the entire intervention system which promotes or hinders adherence to physical activity [15].

The “VISIO-AJUST” study (“Adjust the distance: Better understand the conditions of adherence to videoconference-based programs promoting adapted physical activity during cancer treatments”) was conducted from September 2019 to February 2021. This study was intended for the participants of two videoconference-based programs of adapted physical activity, called “PACTIMe” [16] and “TREVISE” (See programs details in Appendix 1), developed by a French Cancer Center for adult outpatients (before the COVID-19 pandemic period). These interventions were part of the complementary non-pharmacological activities to conventional cancer care proposed by the Department of Supportive Care, in order to contribute to a better quality of life in a global approach through multi-modal supports. “VISIO-AJUST” study aimed to explore the conditions of adherence to videoconference-based programs promoting physical activity during cancer treatment, according to the principles of realist evaluation. In realist evaluation, programs are seen as operating in specific contexts, which interact with the mechanisms by which said programs produce effects [17]. Therefore, what needs to be assessed is “what works, for whom, under what circumstances, and how” [17].

## Methods

### Study design and setting

The VISIO-AJUST study was based on a qualitative successive case approach, guided by the principles of the realist evaluation [18] which belongs to the Theory-Driven Evaluations paradigm [15]. Each “case” referred to a participant considered in a given context.

The results of the VISIO-AJUST study are presented following the RAMESES II reporting standards for realist evaluations [17] (see Appendix 2).

### Description of interventions

The PACTIMe and TREVISE programs included the most recent expert recommendations for medical practitioners to promote physical activity as an integral part of the treatment of chronic diseases [12]. They use the same educational methods applied by the same professionals with patients followed by the same team of caregivers. They were entirely run remotely thanks to the use of a secure web platform (VisioMoov®) developed by Mooven, which is a socially responsible company specializing in health problematics and delivering adapted physical activity sessions by videoconference. These interventions integrated weekly, professionally supervised, adapted physical activity sessions and a set of health workshops in small groups (intended to help patients in the daily self-management of symptoms and facilitate long-term behavior change), supported regular autonomous physical activity (self-monitoring facilitated by the completion of a daily logbook), and combined close individualized supervision (entry and final fitness assessments, follow-up phone calls, and e-mail exchanges making possible a tailored and flexible progression plan) with a collective framework (enabling peer support) to optimize the motivation to regular practice, despite the physical and psychological limitations that may arise.

The PACTIMe program targeted immune therapy-related fatigue and lasted 6 months. Its feasibility and acceptability were evaluated with a prospective longitudinal quantitative approach (full details are provided in [19]). The TREVISE program, initially designed for patients with colorectal cancer during their chemotherapy to prevent deconditioning, was widely opened to patients at the first national lockdown, regardless of the type of cancer and treatment phase. In this program, patients could be accompanied for 3, 6, or 9 months, depending on their needs; health workshops were optional.

### Theoretical framework

Digital healthcare practices have introduced a new dimension in the relationship between patients, relatives, and health professionals, however, this phenomenon has not been adequately explored. The realist evaluation has been proven particularly appropriate to evaluate complex interventions, by considering dynamically the interventions, the contexts in which they are implemented and which influence outcomes, and the non-linear interactions between interventions and contexts [20]. Its methodology proposes a mechanistic interpretation of the process of change and the production of outcomes, by defining and testing causal chains (ordered sequence of events) which are expressed in terms of *“Context-Mechanism-Outcome”* (CMO) configurations [15, 17]. In sum, context refers to two different elements: (i) those which constitute the external environment (Ce) of the intervention (spatial, temporal, political, socio-economic, cultural, individual, etc.), (ii) those which are specific to and introduced by the intervention (Ci) [21]. All these elements (Ce + Ci) may have an impact on the outcomes by triggering or hindering mechanisms. We used the theoretical domains described by S. Michie [22] to help us identify the activated mechanisms [23]. At last, outcomes designate all the potential effects resulting from the interactions between context and mechanisms [15].

**Table:** 

These mechanisms are defined as follows: - “an element of reasoning and reaction of an agent with regard to an intervention productive of an outcome in a given context” (e.g., feeling more self-confident or more motivated) [21, 24]. - as the processes by which an intervention regulates behavior [25]	

### Data collection

A 3-step process was performed to bring out key informant CMO configurations, which could influence the conditions of patient adherence to the programs. In this study, the *conditions of adherence* encompassed the factors likely to influence the *initiation* of the intervention, as well as the patients’ *attendance* and *retention* for the duration of the remote intervention, whether in a positive or negative sense. For this process, the study protocol was approved by the French Protection to Persons Committee (N°ID-RCB 20 2020-A00021-38).

*Step 1: identification of factors likely to influence conditions of patients’ adherence* in the context of remote adapted physical activity programs, by combining data from literature and stakeholders’ knowledge and experience. To this end, the research team conducted a scoping review (see Appendix 3), two independent seminars (the first one including professionals involved in the conception, implementation, or management of the PACTIMe and TREVISE programs; the second one including health professionals, exercise physiologist, and representatives of patients), and a first set of 8 in-depth open-ended phone interviews with participants who were approached in the order of their registration in programs (whatever the program).

*Step 2: elaboration and testing of explanatory CMO configurations* based on a second set of 20 semi-structured phone interviews conducted with participants of the programs by a research engineer. Interviews were audio recorded and professionally transcribed. He did not collect any new data from the 16th interview (data saturation) and the 4 additional interviews enabled us to check that we had indeed reached data saturation. The interview guide was detailed from the first step of the interviews (see Appendix 4). Patients were invited to participate by the study coordinator in the order of their registration in the programs until data saturation was reached, that is, until no new information emerged. The pandemic context also forced us to modify the research protocol, by not conducting two interviews with each interviewee.

*Step 3: refinement of CMO configurations* with a final seminar organized by the research team with some of the professionals involved in the conception, implementation, or management of the PACTIMe and TREVISE programs, and some of their participants who have been voluntary and available. The seminar started by discussing the main contexts involved in program adherence among all those already identified to complete, eventually modify, and validate them in groups. Then, the mechanisms with which they were associated were discussed in the same way.

### Data analysis

During step 1, a detailed report of each seminar was produced. The two seminars were held on July 17, 2020, and December 06, 2021. A number of potential factors likely to influence the conditions of patients’ adherence to programs were identified, by crossing data from the scoping review, the seminars, and the first set of interviews. To cross-reference the data, the results of the scoping review were used as a basis for the seminar. Participants were able to validate the data collected in the literature according to their knowledge of their particular field. The first interview grid was then constructed on the basis of these results.

During step 2, all interviews were fully audio recorded and professionally transcribed verbatim for in-depth analysis. A thematic content analysis [26] was performed on the transcripts using NVivo 12 Software by the research engineer and a second independent researcher to check for consistency [27]. A cross-sectional analysis was then carried out to identify the recurrence of the Context-Mechanism-Effects (CMO) configurations through all cases.

Simple statistics were performed to describe the demographic and medical characteristics of the study participants (directly collected from them by the study coordinator).

During step 3, a detailed report of the final seminar was written, including what had been particularly discussed or supported.

The results of each step were supervised and discussed with an expert in realist evaluation

## Results

### Step 1: identification of factors likely to influence conditions of patient adherence

External main factors to the program likely to influence the conditions of patients’ adherence are related to oncologists, as well as the patient himself, his environment, and his peers. They are categorized in the form of a table (see Appendix 5). Concerning the program, the factors are related to the modalities of entry (e.g., prior information), the modalities of progress (e.g., adaptation and progressiveness according to each person, free of charge), its perceived quality (e.g., perceived competence of the coach), during the treatment or not (see details in Appendix 6).

### Step 2: elaboration and testing of CMO configurations

#### Description of participants

Twenty interviews were conducted during this step. The main participants’ demographic and medical characteristics are described in Appendix 7.

The mean age of participants was 51 years. The majority of included participants were women with gynecological cancer (breast, ovarian, uterine). Slightly more than half of the participants had metastatic cancer and were undergoing treatment. The most represented treatments were immunotherapy, chemotherapy, and targeted therapy. Sixteen persons were participants from the TREVISE program and four from the PACTIMe program.

#### CM configurations associated with the adherence to programs (O)

Five main CMO configurations emerged from the analyses. Key quotes in the sidebar illustrate each configuration.

1. An accessible resource and a favorable environment for the practice of adapted physical activity

Mechanism 1 (M1): Perceive programs as an accessible resource and a favorable environment.

Contexts that activate this mechanism:

- Individual context is marked by the need to be guided in order to practice physical activity (Ce),
- Absence of adapted (Ce36) and financially accessible support structures close to the home (Ce),
- Offering regular physical activity sessions (Ci),
- Taking part in a small group (Ci),
- Encountering people who have the same disease without having the same physical condition (Ci),
- Supervision by a trained and specialized teacher (Ci) able to adapt the exercises to the capacities of the participants (Ci),
- Friendly and benevolent climate, with the support of a playful pedagogy (Ci),
- Practice of an adapted physical activity becomes compatible with the organizational constraints of daily life,
- Accessible to the participants who can follow the sessions by videoconference (Ci) from their home,
- Accessible without having to move (Ci),
- Accessible while protected from the risks of contamination (Ci).

Contexts unfavorable to the outcome:

The access to the programs and the quality of the follow-up of the sessions are nevertheless contingent on the possession of functional computer equipment and an Internet connection (Ce), which can ensure good visibility and communication.

Key quote

**Table:** 

“The fact that I had to make long trips limited my physical activity enormously, since I had difficulty motivating myself, you know, sometimes there are days when you don't feel like it, and the fact that it's in video, you remove a number of important constraints, you don't really need to prepare beforehand, it's enough to take five minutes before putting on your sports clothes and turning on your computer”.

2. A setting conducive to sociability despite distances

Mechanism 2 (M2): Find a source of sociability (M2).

Contexts that activate this mechanism:

- A feeling of social isolation (Ce),
- Interacting with other people who have a similar medical situation (Ce),
- The need to be accompanied during physical activity (Ce),
- Establishing a link with a teacher:
  - ◦ whom they consider competent, sympathetic, and a good listener (Ci),
  - ◦ who structures the time for speaking while allowing it to circulate between participants (Ci),
  - ◦ who guarantees of a benevolent framework (Ci)
- Be able to take part in the exchanges of their group (Ci),
- Belonging to a relatively fixed group of people (Ci) with whom they can compare their state and their progress without a spirit of competition, and find support in the most difficult moments.

Contexts unfavorable to the outcome:

The visual limits of the videoconference sometimes interfere with the fluidity of the exchanges. However, they do not constitute a major obstacle to the creation of links (Ci).

Key quote

**Table:** 

“We motivate each other. We see that there are some who can do certain movements, others not. So [...] we say to each other, 'I can't do it, I'm not alone. I don't feel inferior. We are all on the same scale. And then we all suffer from the same pathology. So it strengthens the bonds to know that we are all in the same boat but that we manage to move a little once a week with a coach who takes us in hand.”

3. Building confidence and securing practices

Mechanism 3 (M3): Feel of comfort and reassurance

Contexts that activate this mechanism:

- Staying in the familiar setting of the home (Ci),
- Benefiting from the friendly and benevolent climate of group physical activity sessions (Ci),
- A referent teacher is appreciated for his professional qualifications and personal qualities (Ci),
- Regularity of the schedules and duration of the sessions (Ci),
- Adapting exercises to the possibilities of each one, according to his physical condition of the day (Ci),
- Belonging to a relatively fixed group of people (Ci) without fear of the glance and the judgment of others.

Contexts unfavorable to the outcome:

- Lack of adaptability of the program (days and times of the sessions (Ci)
- Program length is considered too short and not adaptable (Ci)
- Lack of adaptability of the exercises (Ci)
- Changing teachers during the program (Ci)
- Perceiving heterogeneity between participants in the same group in terms of physical abilities (Ci).

Key quote

**Table:** 

“And we had a private class, we'll say with a coach who was on the other side of the screen and who showed us the movements, who counted with us, who gave us tricks that allowed us to do movements that we couldn't do otherwise in other positions so that we could still move. And then always with lots of motivation, lots of little encouragements, very human things, we'll say”

4. A combination of several motivational levers in favor of continuous effort and progress

Mechanism 4 (M4): Support motivation

Contexts that activate this mechanism:

- Small group sessions (Ci),
- Fixed session times and days (Ci),
- Supervision quality (Ci),
- Contributing to the friendly and benevolent atmosphere (Ci),
- Varied exercises (Ci)
- Adapted exercises (Ci)
- Playful spirit of the exercises (Ci)

Key quote

**Table:** 

“So it helps me live. It allows me to see that I can still do my movements despite the pain. [...] Physically, it's rewarding and I tell myself that I'm not ruined. And then psychologically, it's a great support because it's a little daily appointment, well not daily but [...] weekly. It's a bit like having an appointment with a girlfriend to chat. Except that we don't chat, we do sports.”

5. Regularity of sessions, progressiveness in the effort, and evaluation of progress as a basis for the adoption of a regular physical practice

Mechanism 5 (M5): Perceive positive consequences on one's health (physical and mental state) (M5) and mechanism 6 (M6): Progressive integration of a routine

Contexts that activate this mechanism:

- Fixed rhythm and the approximation of the sessions (Ci),
- Accompaniment of a professional in the effort (Ci),
- Adapting the exercises to the possibilities of each person (Ci),
- Supporting by individualized advice on how to practice without putting oneself in difficulty (Ci),
- Developing on the basis of evaluations regularly carried out, in a short way at the beginning and end of each session (Ci),
- Evaluating in a more in-depth way during individual progress points (Ci).

Key quote

**Table:** 

“The positive thing is to be able to feel a progression, that's also positive [...] that is to say that after a while you feel that you can do the exercises better than at the beginning, you can do them more times, I just did, at the end of the six months there is an evaluation to see the progression between before and after, and here, for me we could clearly see that there was an improvement of the physical condition. So I think it's also motivating, it's a pleasure to realize that, well, it works.”	

#### Step 3: refinement of CMO configurations

No factor could be prioritized by the group. All factors were considered equally important. Rather, the key factors vary from one individual to another depending on many individual characteristics, which also change over time, for example, in relation to the person's time in treatment or how long they have been in the program. Moreover, no modifications or new factors were added.

## Discussion

The main objective of the VISIO-AJUST study was to explore the conditions of adherence to a remote device aimed at promoting physical activity during cancer treatment, based on the experience of patients who participated in the PACTIMe and TREVISE programs delivered by videoconference, and according to a realistic evaluation methodology. The analysis of associations between contextual factors (C), mechanisms (M), and effects (O) highlighted that both the PACTIMe and TREVISE programs facilitated access to (1) friendly and benevolent environment; (2) secure with professional supervision and adaptable to differences in individual situations; (3) sufficiently motivating and structuring to support continuity and progressiveness in the effort. These results confirm that the PACTIMe and TREVISE programs were able to remove a certain number of known barriers to the practice of physical activity [5], by acting on the physical and social environment of the patient. For video-conference-based interventions, it is the intervention that goes to the patient and not the reverse: this makes the practicing of physical activity accessible, through the easy insertion of the program into the protective and daily environment of the home. The search for a shared experience with a group of peers, present in many of the participants, also found a favorable resonance in the collective format, without the distance being an obstacle to the emergence and installation of a real group dynamic, uniting the participants in emulation and common progress. It is remarkable that despite the distance and certain visual limitations reported by the participants, the conditions of experience sharing and social support were met; we observe effects such as the reinforcement of the feeling of competence and belonging, as described and sought in group interventions as catalysts of change [28, 29].

It is also noteworthy that the adapted physical activity teacher was clearly designated as an essential actor in facilitating links, as well as in supervising the exchanges, thus ensuring the conviviality of the setting and the neutrality of judgment. In this role, his qualifications and personal qualities were emphasized by many participants. The professionalism of the supervisor, particularly regarding the ability to connect with the group on a relational level, was also vocalized by the participants as important for the teacher’s capacity to propose exercises adapted to the physical condition of each person while designing the group sessions. In short, the participants' feeling of commitment, which appeared to be one of the mechanisms involved in attendance, seems to have been mobilized as much towards the peer group as towards the teacher. The influence of family and friends, as an external contextual factor and support for adherence, occupied a much more modest place in the participants' discourse, even though it was cited in the latest INSERM expert report on physical activity in chronic illness as an essential source of motivation for effective practice [12]. An explanatory hypothesis for this lesser influence could be that in the PACTIMe and TREVISE programs, the group dynamic, including the relationship with the teacher, took precedence and was sufficient in itself without the need for any other form of social influence, including that coming from health professionals during these programs, which was completely absent from the participants' discourse.

In line with the recommendations of the INSERM report [12], Among the support methods identified as being most conducive to adherence to the PACTIMe and TREVISE programs, the fixed framework (rhythm, schedule, duration, structuring of sessions) proved to be as much linked to the security of the participants, by providing stable reference points, as to their commitment to an activity, which could also in this way become “routine”, in the sense of a life habit. In this respect, the “appointment” effect proved to be a strong lever of adhesion and behavioral change, taking into account the regularity of the sessions as well as the consideration for others and the desire to continue being part of the group. The differences in physical condition between participants also emerged as one of the motors of the social influence mechanism favoring reassurance, the reinforcement of the feeling of competence, and the motivation to progress: several participants indeed clearly indicated that by comparing themselves to others, weaker or on the contrary more in shape, they could situate themselves by finding the courage to hang on in the effort.

The sessions and the quality of the interactions did not appear to suffer too much from intermittent technical difficulties related to the instability of the connections, probably thanks to the technical assistance provided by the teachers to the participants who were less familiar with videoconferencing and to the mutual help between participants during the sessions. However, the PACTIMe and TREVISE programs were not able to fully address the inequalities in Internet access and the material disparities that affect participation in distance interventions. To do so, it would have been useful to be able to offer a loan of a tablet or a free Internet subscription, which unfortunately was not feasible for financial and logistical reasons (sending/installation/maintenance of the equipment). Another basic problem was access to information: the interviews with participants in the PACTIMe and TREVISE programs highlighted the inadequate circulation of information about existing resources, with a particular lack of relay in the community of health professionals, including oncologists whose lack of communication on lifestyle recommendations is well known [30]. Regardless of the digital issues, there is still a need to effectively integrate interventions aimed at promoting health behaviors into the healthcare system, and to find levers, particularly organizational ones, to ensure that more patients have access to resources that have been shown to be beneficial to their health. Finally, although the pandemic situation obliged us to forego two interviews with each person, the results of the study nevertheless highlighted the persistence of a lack of access to information, partly linked to the attitude of health professionals who would be in the front line in directing patients to the appropriate resources. This theory-based evaluation modality, the realist evaluation, has kept its promise of making it possible to report on the key elements mobilized according to the interventional or external contexts that may have favored patients' physical activity practices. In this approach, the mobilization of all the stakeholders makes it possible to identify the real key elements that promote adherence to the intervention. The validation of these key elements by the participants assures their quality.

## Study’s limitations

In this study, it was not possible to investigate the reasons for non-participation in the programs (both dependent and independent of patient choice), other than those related to equipment and Internet access problems, as well as to a rapid deterioration of the general state of health requiring an early interruption of program participation, since only patients who participated in the programs could be interviewed. The specificities related to the different confinements also influenced participation in the program. Indeed, this remote program may have appeared to be the only possibility for some patients to benefit from a social life.

Because of the epidemic context, we were only able to interview one participant at a time, and therefore the information collected on the conditions for continuing regular physical activity after the programs remained fragmentary. However, we were able to verify that the programs mobilized a wide variety of mechanisms favorable to behavioral change, thus increasing the probability that participants would continue to engage in physical activity after their participation in one or other of the programs.

Finally, while Context-Mechanism-Effect (CMO) configurations have the advantage of being able to convey a synthetic view of the complexity of the interactions that drive a phenomenon, such as program adherence, they also have the limitation of simplifying some of the factor relationships studied and reducing their overall scope. While this study brings key elements of distance program design to the attention of professionals, further study will further refine the results.

## Conclusion

In the context of the exponential development of videoconferencing, this original study questions the assumption that digital technology will overcome the barriers to physical activity in cancer patients undergoing treatment. This study shows that it is only under certain conditions that remote programs of physical activity can effectively remove some of the barriers to regular practice, in particular by acting on the physical and social environment of the patients. Further research with a longer follow-up would help explore which key conditions better promote sustainable behavior changes.

### Supplementary Information

**Additional file 1:** **Appendix 1.** PACTIMe and Trevise programs.

**Additional file 2:** **Appendix 2: Table S1.** Checklist of RAMESES II reporting standards for realist evaluations.

**Additional file 3:** **Appendix 3.** Scoping Review.

**Additional file 4:** **Appendix 4: Table S2.** Interview grid.

**Additional file 5:** **Appendix 5: Table S3.** Factors related to oncologists likely to influence conditions of patients’ adherence. Table S4. Factors related to the patients’ social environment likely to influence their conditions of adherence.

**Additional file 6:** **Appendix 6: Table S5.** Quotes from participants to illustrate each CMO configuration.

**Additional file 7:** **Appendix 7: Table S6.** Main participants’ demographic and medical characteristics.

## Data Availability

The datasets used and/or analyzed during the current study are available from the corresponding author upon reasonable request.

## Abbreviations

C: Context
Ce: External context
Ci: Interventional context
M: Mechanism
O: Outcome
CMO: Context-Mechanism-Outcome
