# Supplementary material for: Conditions for adherence to videoconference-based programs promoting adapted physical activity in cancer patients: a realist evaluation
Source: Implement Sci. 2024 Jan 29;19:6. doi: 10.1186/s13012-024-01338-y (PMC10823602; doi:10.1186/s13012-024-01338-y)
Supplement: Supplementary file 1 — Additional file 1: Appendix 1. PACTIMe and Trevise programs. [file 13012_2024_1338_MOESM1_ESM.docx]

**APPENDIX 1**

**PACTIME and TREVISE PROGRAMS**

**TREVISE**

The TREVISE project was part of a preventive videoconferencing approach, targeting the risk of physical deconditioning. It offered support in three stages, with patients able to choose to commit to a period of one, two or three trimesters. Patients could participate in up to two group physical activity sessions per week via videoconferencing. The encouragement of additional unsupervised physical activity of increasing duration throughout the program, and the construction of a personalized orientation plan in the final quarter, were designed to ensure gradual, secure and confident autonomy, and the lasting integration of health recommendations. The professionalism and regularity of the follow-up (supervised by an adapted physical activity teacher) were justified by the fragile state of patients during the treatment period. The integration of informative and practical workshops (such as "nutrition and cancer") was based on the desire to open up care beyond the strictly oncological field, to encompass health and life paths, and thus ease the transition between treatment and post-treatment phases. The TREVISE program was made available free of charge thanks to the financial support of the Gustave Roussy Foundation.

**PACTIME**

The PACTIMe project pursued a therapeutic objective of reducing fatigue under immunotherapy, by offering a 6-month combination of weekly adapted physical activity (APA) sessions and workshops to help manage fatigue through informative and educational inputs, based on motivational and cognitive-behavioral techniques [6]. The use of videoconferencing is particularly useful in the disabling context of immunotherapy-associated fatigue. Among the obstacles to taking part in complementary care activities, physical limitations are often compounded by the long intervals between injections and hospital visits - intervals during which patients can find themselves relatively isolated.

**Among the points that distinguished them from interventions already developed in oncology on the "connected" model (mobile applications, web portal...), these programs had in common:**

- Direct medical referral of patients, with a certificate of no contraindication to the practice of APA as a condition of enrolment, following a medical examination,

- Ensuring safe practice by integrating an initial, individual assessment of patients' physical condition, thanks to environmental tests carried out by the qualified APA teacher (who is also responsible for supervising APA sessions); this assessment is repeated after six months,

- An "embodied" interactive dimension, in the sense that the use of videoconferencing enabled direct exchanges and visual contact between patients and the APA teacher, or between patients during APA sessions,

- A combination of group benefits (social support, vicarious learning, etc.) and individual benefits (personalized follow-up, taking account of personal circumstances and in particular specific difficulties/fragilities).
