# Supplementary material for: Conditions for adherence to videoconference-based programs promoting adapted physical activity in cancer patients: a realist evaluation
Source: Implement Sci. 2024 Jan 29;19:6. doi: 10.1186/s13012-024-01338-y (PMC10823602; doi:10.1186/s13012-024-01338-y)
Supplement: Supplementary file 2 — Additional file 2: Appendix 2: Table S1. Checklist of RAMESES II reporting standards for realist evaluations. [file 13012_2024_1338_MOESM2_ESM.docx]

**APPENDIX 2**

**Table 1: Checklist of RAMESES II reporting standards for realist evaluations**

| TITLE |  | Y/N/Unclear | Page(s) in manuscript |
| --- | --- | --- | --- |
| 1  SUMMARY OR ABSTRACT  2 | In the title, identify the document as a realist evaluation  Journal articles will usually require an abstract, while reports and other forms of publication will usually benefit from a short summary. The abstract or summary should include brief details on: the policy, programme or initiative under evaluation; programme setting; purpose of the evaluation; evaluation question(s) and/or objective(s); evaluation strategy; data collection, documentation and analysis methods; key findings and conclusions  Where journals require it and the nature of the study is appropriate, brief details of respondents to the evaluation and recruitment and sampling processes may also be included. Sufficient detail should be provided to identify that a realist approach was used and that realist programme theory was developed and/or refined | Y  Y | 1  2 |
| INTRODUCTION  3 Rationale for evaluation  4 Programme theory  5 Evaluation questions, objectives and focus  6 Ethical approval | Explain the purpose of the evaluation and the implications for its focus and design  Describe the initial programme theory (or theories) that underpin the programme, policy or initiative  State the evaluation question(s) and specify the objectives for the evaluation. Describe whether and how the programme theory was used to define the scope and focus of the evaluation  State whether the realist evaluation required and has gained ethical approval from the relevant authorities, providing details as appropriate. If ethical approval was deemed unnecessary, explain why.  The study protocol was approved by the French Protection to Persons Committee (N°ID-RCB 2020-A00021-38) | Y  Y  Y  Y | 4  5  5  8 |
| METHODS  7 Rationale for using realist evaluation  8 Environment surrounding the evaluation  9 Describe the programme policy, initiative or product evaluated  10 Describe and justify the evaluation design  11 Data collection methods  12 Recruitment process and sampling strategy  13 Data analysis | Explain why a realist evaluation approach was chosen and (if relevant) adapted  Describe the environment in which the evaluation took place  Provide relevant details on the programme, policy or initiative evaluated  A description and justification of the evaluation design (i.e. the account of what was planned, done and why) should be included, at least in summary form or as an , in the document which presents the main findings. If this is not done, the omission should be justified and a reference or link to the evaluation design given. It may also be useful to publish or make freely available (e.g. online on a website) any original evaluation design document or protocol, where they exist  Describe and justify the data collection methods – which ones were used, why and how they fed into developing, supporting, refuting or refining programme theory  Provide details of the steps taken to enhance the trustworthiness of data collection and documentation  Describe how respondents to the evaluation were recruited or engaged and how the sample contributed to the development, support, refutation or refinement of programme theory (Note : No sampling in this research)  Describe in detail how data were analysed. This section should include information on the constructs that were identified, the process of analysis, how the programme theory was further developed, supported, refuted and refined, and (where relevant) how analysis changed as the evaluation unfolded | Y  Y  Y  Y  Y  Y  Y | 7  6  6  7  8  8  9 |
| RESULTS  14 Details of participants  15 Main findings | Report (if applicable) who took part in the evaluation, the details of the data they provided and how the data was used to develop, support, refute or refine programme theory  Present the key findings, linking them to contexts, mechanisms and outcome configurations. Show how they were used to further develop, test or refine the programme theory | Y  Y | 10  10-11-12-13-14 |
| DISCUSSION  16 Summary of findings  17 Strengths, limitations and future directions  18 Comparison with existing literature  19 Conclusion and recommendations  20 Funding and conflict of interest | Summarise the main findings with attention to the evaluation questions, purpose of the evaluation, programme theory and intended audience  Discuss both the strengths of the evaluation and its limitations. These should include (but need not be limited to): (1) consideration of all the steps in the evaluation processes; and (2) comment on the adequacy, trustworthiness and value of the explanatory insights which emerged In many evaluations, there will be an expectation to provide guidance on future directions for the programme, policy or initiative, its implementation and/or design. The particular implications arising from the realist nature of the findings should be reflected in these discussions  Where appropriate, compare and contrast the evaluation’s findings with the existing literature on similar programmes, policies or initiatives  List the main conclusions that are justified by the analyses of the data. If appropriate, offer recommendations consistent with a realist approach  State the funding source (if any) for the evaluation, the role played by the funder (if any) and any conflicts of interests of the evaluators | Y  Y  Y  Y  Y | 15  16-17-18  16-17  19  22 |
