# Supplementary material for: Conditions for adherence to videoconference-based programs promoting adapted physical activity in cancer patients: a realist evaluation
Source: Implement Sci. 2024 Jan 29;19:6. doi: 10.1186/s13012-024-01338-y (PMC10823602; doi:10.1186/s13012-024-01338-y)
Supplement: Supplementary file 3 — Additional file 3: Appendix 3. Scoping Review. [file 13012_2024_1338_MOESM3_ESM.docx]

**APPENDIX 3**

**Scoping Review (not published)**

**Factors of adherence relative to different types of interventions proposed to cancer patients using a digital support and integrating physical activity**

**Introduction**

This literature review focuses on the use of connected (digital) devices to promote physical activity in oncology (during or after treatment). In this context, we conducted a scoping review, which analyzes the key concepts underlying a field of research, highlighting theories, main sources and types of results available.

The main objective of this review was to identify factors of adherence relative to different types of interventions proposed to cancer patients using a digital support and integrating physical activity. The secondary objectives of the review were to identify factors present in the interventions described as effective, and also to explore different devices existing in the literature: their target population, their objectives, digital supports used...

This scoping review is therefore broader than adherence to the programs that interest us in this article. We will therefore detail the method in its entirety, but in the results, we will be particularly interested in adherence and the use of digital devices (see part d. of the results).

**Method**

We based our methodology on the PRISMA Extension for Scoping Reviews (PRISMA-ScR) checklist (1).

Inclusion criteria were as follows: 1) Empirical/original studies, 2) Conducted in oncology, 3) Involving adult patients, 4) In treatment or post-treatment phase, 5) Dealing with physical activity (means or purpose), 6) Use of an online device 7) As part of a program (or potential program). The exclusion criteria were, in order: 1) Type of article: systematic or non-systematic literature reviews, meta-analyses, conference abstracts, study protocols, erratum, letters 2) Study population without cancer: general population, caregivers 3) Pediatric population 4) No reference to physical activity 5) No use of an online device: face-to-face interviews, DVDs, phone calls, sms/text messages, used alone, connected devices without structured content delivery (used alone as measurement tools) 6) General physical activity studies on connected media outside the framework of a program (or potential program).

We used the "PubMed" and "Scopus" databases for our bibliographic search.

The search was based on the following equation: "cancer" AND ["exercise" OR "physical activity"] " AND ["web-based" OR "internet" OR "online" OR "digital" OR "mHealth" OR "telemedicine)"], considering references published in French and English, between 2015 and 2019. To select these keywords, we searched the databases for keywords used in articles corresponding to our reference criteria. We selected keywords that appeared several times, putting in more synonyms concerning connected approaches, as this was the characteristic we wanted to emphasize.

Articles were selected in three stages: first on title to remove duplicates and articles not corresponding to the inclusion criteria, then on abstract and finally on full text to refine the search and remove articles not meeting the inclusion criteria.

To extract the data required for our research, we designed an extraction grid in pairs. This grid lists the following information:

- Type of article

- Study design

- Characteristics of the population studied: type of cancer, age, sex, treatment phase, country of study and number of participants;

- A description of the intervention: type of connected support used, combinations with other devices or strategies (contact with professionals during the program, collective dimension of the program or face-to-face elements), duration of the intervention, and type of physical activity promoted.

- A description of control measures (comparator arms where appropriate);

- Study objectives: primary objective, secondary objectives and measures employed;

- Results in terms of adherence, acceptability and efficacy of the intervention;

- Factors associated with adherence, acceptability and effectiveness;

- Methodological strengths and limitations of the studies;

- The theoretical models used;

- Consideration of digital access as a factor of inclusion or exclusion.

For the purposes of this literature review, we did not make any selection on the basis of the methodological quality of the studies, in order to gain an overview of the different systems and thus favour a broad approach. We did, however, exclude literature reviews, research protocols, conference abstracts, errata and letters, as these documents do not provide information on the conditions of enrolment.

**Results**

- 1. Selection of articles

A search of the PUBMED and SCOPUS databases identified 836 references. A first reading of the titles led to the exclusion of 708 references, 176 of which were duplicates. After consulting the abstracts, we retained 65 articles, which we read in their entirety. Of these, 8 did not fully meet the inclusion criteria. In the end, we drew up a sample of 57 articles. Figure 1 shows the main stages in the selection process (see Figure 1 below: Flow-Chart).

Figure 1: Flow-Chart


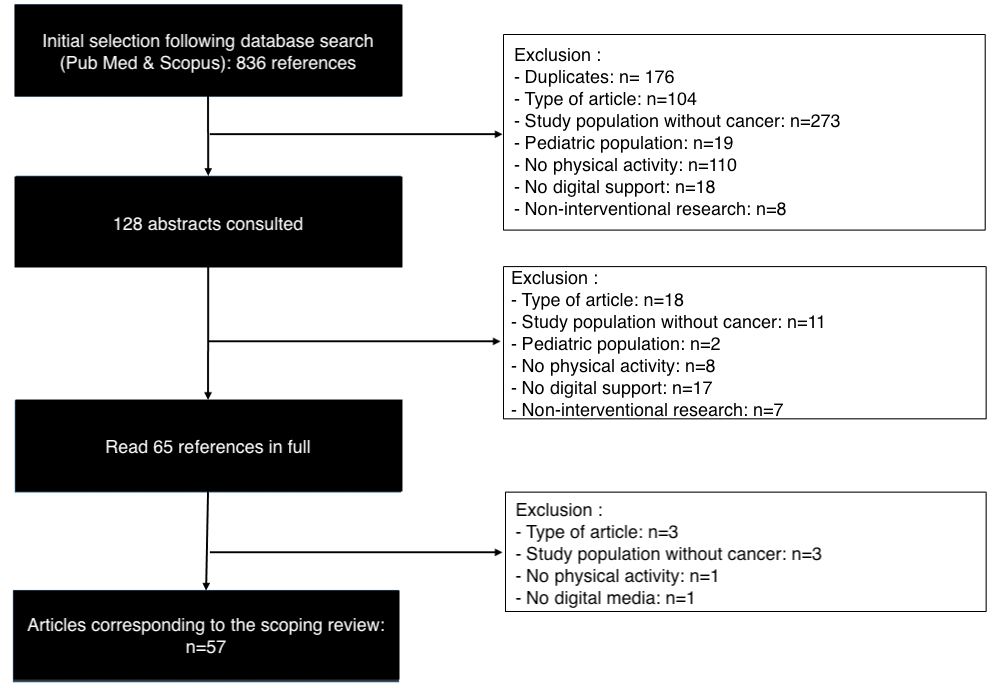


- 1. Description of selected studies
     1. Design

Of the 57 articles selected, 30% (n=17) were pilot studies, 39% (n=22) randomized controlled trials and 9% (n=5) uncontrolled randomized trials. Vast majority of studies (86%, n=49) adopted a longitudinal approach. Methods employed were predominantly quantitative, with only 14% (n=8) mixed studies and 11% (n=6) qualitative studies.

- - 1. Study population

Study populations were predominantly American (40%, n=23), Dutch (16%, n=9), Australian (11%, n=6) and Korean (11%, n=6). Concerning the number of patients included in the studies, 44% (n=25) of articles reported fewer than 50 patients, 26% (n=15) between 50 and 100 patients, 16% (n=9) between 100 and 300 patients and 14% (n=8) more than 300 patients.

In terms of pathologies and treatments, the three most common types of cancer were breast (68%, n=39), genitourinary (37%, n=21) and digestive (33%, n=19). More than two-thirds of the studies (70%, n=40) focused on the post-treatment period, 25% (n=14) on the treatment period, and only 5% integrated the during-treatment and post-treatment phases.

- - 1. Therapeutic goals

Of the interventions studied, 44% (n=25) had the primary objective of increasing physical activity levels. In some programs, the physical activity objective was coupled more with health behavior adoption objectives (including areas such as physical activity, nutrition, sleep, social relationships, emotion management, alcohol and tobacco) (16%, n=9). In the other studies, physical activity was used more as a means, rather than a goal, to achieve various therapeutic objectives, such as improving quality of life (16%, n=9), weight loss (11%, n=6), preventing physical deconditioning (9%, n=5) or reducing pain (5%, n=3).

- - 1. Physical activity measurements

Just over half the studies included a measure of patients' physical activity level (54%, n=31). Of these, 65% (n=20) used self-report questionnaires, 29% (n=9) used automatic activity recording tools (e.g. pedometers) and 6% (n=2) combined both forms of measurement. In terms of self-report questionnaires, the most widely used among validated tools were the International Physical Activity Questionnaire Short Form (IPAQ) (27%, n=6), the Godin Leisure-Time Exercise Questionnaire (GLTEQ) (23%, n=5) and the Self-report Short Questionnaire to Assess Health Enhancing Physical Activity (SQUASH) (18%, n=4). One-fifth of the studies used ad-hoc questionnaires (18%, n=4).

- 1. Description of intervention studied
     1. Types of intervention

The proposed interventions varied in duration: between 1 and 2 months for 21% (n=12), 3 months for 33% (n=19), or 6 months for 18% (n=10). In terms of the type of media used, the majority of programs were based on a website (56%) or a mobile application (32%). Among website-based programs, 59% (n=19) were solely website-based, while 19% also included a pedometer (n=6), 12% phone calls (n=4), and 10% e-mail reminders (n=3). SMS messages could also be added to these combined devices. Among app-based programs, 28% combined the app with the use of a pedometer (n=5). Through these various connected media, different physical activities were promoted: aerobic and resistance exercises (58%, n=33), walking (19%, n=11), or activities of daily living such as housework or gardening inserted into daily life such as physical commuting (cycling, walking), housework or gardening (11%, n=6).

- - 1. Type of coaching offered

Of the articles analyzed, 49 studied structured programs in particular, while 10 studied preferences and representations around digitally-mediated adapted activity interventions in general. Within the framework of these structured programs, different types of support can be offered, for example, certain professionals can intervene or not, face-to-face or remotely (calls, e-mails...). Here, out of the 49 interventions surveyed, 20% (n=10) set up face-to-face contact with a professional, 23% proposed remote contact (SMS, e-mail, telephone) with professionals, while 43% (n=21) left the patient completely autonomous. Similarly, programs may or may not include a collective dimension, i.e. contact with other patients taking part in the same program, using a forum for example. In this case, of the 47 interventions studied, 20% (n=10) included a collective dimension.

- - 1. Digital access as a factor in inclusion or exclusion

Out of 47 articles studying structured programs in particular, 62% (n=29) of studies explicitly listed access to an internet, computer or smartphone as an inclusion criterion. Of these 47 studies, 47% (n=22) listed not having internet access as an explicit exclusion criterion, and 19% (n=9) listed not having a smartphone as an explicit exclusion criterion.

- 1. Adherence to programs and use of digital devices (focus on the subject of our article)
     1. Results: adhesion and satisfaction

Of the 57 articles analyzed, 57% (n=33) reported adherence to the program. Of these 33 articles, 22 reported an adherence rate of over 80%. Fourteen studies assessed patient satisfaction at the end of the digital intervention, and all of them reported a predominantly positive assessment (using very different measurement tools, making it impossible to assess satisfaction on average).

- - 1. Elements described as facilitating program adherence

None of the studies included a controlled analysis of the factors associated with patient adherence to connected devices. However, a number of them suggest the possible influence of several variables as levers or obstacles. These are most often derived from interviews with patients in mixed or qualitative studies, or more generally from satisfaction questionnaires relating to the use of connected devices. A total of 27 articles mentions factors that may be associated with or promote adherence. Among these factors, it is possible to distinguish, on the one hand, factors relating to the characteristics of the population and, on the other hand, factors relating to the components of the intervention.

Certain individual characteristics are associated with better adherence: young age (2–6), a high level of literacy and education (2,7), and access to the Internet and digital tools (smartphone, tablet, computer) (2,5–8). Certain intervention components would appear to promote adherence to digital physical activity programs. We identified 9 types of factors:

- The provision of content and information perceived as interesting (4,9–17);

- Feedback on physical activity and health outcomes (calorie balance, weight loss, increased physical capacity, etc.) (4,8,11,13,14,18–21);

- The "tailored" dimension of interventions. This so-called "tailored" intervention scheme involves setting up a physical activity program and providing patients with tailored information based on individual characteristics (cancer types, treatment types, age, gender) as well as personal perceptions (health, treatments and physical activity - including baseline physical activity level, appetite for physical activity, barriers/levers and perceived resources). This information can be gathered from telephone or face-to-face interviews, as well as through questionnaires via an app, website or e-mail (8,12,13,15,16,19,22,23);

- Ease of use of the digital device (7,24);

- The provision of learning tools for the use of digital media (website, application, pedometer) (8,19);

- The link with healthcare professionals (4,9,14,16), with contact established face-to-face or remotely via telephone calls, SMS or e-mail;

- Reminders or automatic messages via the digital platform (11,13,20,25);

- Tracking tools, such as a pedometer, especially when connected to the digital platform (15,20,21);

- Social support or experience sharing made possible via the connected platform, with access to a discussion forum for example (13,21,26).

- - 1. Les éléments décrits comme des obstacles à l’adhésion au programme

A total of 22 studies identified factors that could hinder adherence to connected interventions. In the same way as for the elements described as favoring adherence, it is possible to distinguish factors relating to individual characteristics from those relating to intervention components.

Individual characteristics considered to be possibly associated with poorer adherence are advanced age (2,3,6,13), being male (23,27), low income (23), not having access to the internet or digital equipment (computer, smartphone, tablet) (2,6,8,13,15,16,28). The incidence of treatment and illness is also highlighted as an important barrier. Among intervention-related factors, we identified the following as the main obstacles:

- Problems related to the use of the digital device (difficulty connecting, using, finding content, etc.) (5,9,16,21,29,30);

- The time-consuming aspect of the program, too great an effort required, too regular a connection required, or too much information to be filled in (5,10,16,21,25,31,32);

- Information deemed by patients to be inappropriate, confusing, irrelevant or contradictory (15,30);

- Lack of contact with healthcare professionals (4,17).

- 1. Effectiveness of interventions on patients' physical activity levels
     1. Results

Of the 25 studies in which the aim of the intervention was to increase physical activity, 14 (56%) assessed its effectiveness. Of these 14 interventions, 7 (50%) were described as significantly effective. None of the studies carried out a controlled analysis of the factors associated with the efficacy or inefficacy of the proposed intervention. Nevertheless, it is possible to identify a number of factors that might be involved, based on the authors' suggestions.

ii. Characteristics of effective interventions

Of the 7 studies described as significantly effective (increase in physical activity), 4 used an app, 3 used a website. The combined use of a connected pedometer and a digital platform was found in 4 programs.

Ease of use of the digital platform (24,33) feedback (4,21) and the possibility of exchanges between participants from the connected platform (21,33) were features relatively common to these programs. Although mentioned only once, telephone counseling sessions to complement the digital platform (4), and tailored interventions (34) were also among the factors that could have a positive influence on physical activity.

**Conclusion:**

**TREVISE and PACTIMe: promising programs in terms of adherence**

The literature review we conducted enabled us to identify nine program-related characteristics described as promoting cancer patients' adherence to connected adapted physical activity devices. Of these nine factors, eight are present in the TREVISE and PACTIMe programs. The following table presents the factors relating to interventions described in the literature as promoting patient adherence, and shows how these factors are present in the TREVISE and PACTIME programs.

| **Intervention-related factors described in the literature as promoting adherence** | **Presence of these factors in the TREVISE and PACTIME programs** |
| --- | --- |
| **The provision of content and information perceived as interesting**  (4,9–17) | The TREVISE and PACTIMe programs provide information to patients through motivational interviewing during the initial physical check-up, as well as through informative and practical workshops on physical activity, diet and fatigue in oncology. |
| **Feedback on physical activity and health results**  (4,8,11,13,14,18–21) | PACTIME and TREVISE programs provide feedback to patients, since after each group session, a review of the session is made with the adapted physical activity teacher. In addition, telephone calls with the adapted physical activity teacher enable patients to provide feedback on their progress and motivation in the program, and help identify any difficulties. |
| **The "tailored" dimension of the program**  (8,12,13,15,16,19,22,23) | The TREVISE and PACTIMe programs include a " tailored " and personalized dimension. Physical assessment at the start of the program enables us to discuss with each patient his or her physical activity habits, motivational factors and any obstacles to regular exercise. This personalized assessment enables us to adapt individual sessions to the specific characteristics of each patient. For example, if a patient has mobility problems with certain limbs, the physical activities set up will propose gentle use of these limbs. |
| **Ease of use of the digital device**  (7,24) | TREVISE and PACTIME programs are based on the V@Si platform that has already been used, although the programs, platform functionalities and populations are different. |
| **Setting up learning tools for the use of digital media**  (8,19) | TREVISE and PACTIME programs offer training in the use of the platform. During the initial physical check-up, participants will be shown how to use the digital platform, which will be used to supervise physical activity sessions and facilitate exchanges between adapted physical activity teachers and participants. |
| **A link with healthcare professionals**  (4,9,14,16) | TREVISE and PACTIME programs ensure this link between professional and patient by offering regular support from different healthcare professionals throughout the program. This support is illustrated by the physical activity sessions supervised by an adapted physical activity teacher, the cycle of workshops on physical activity, fatigue and nutrition led by professionals qualified in these fields, and the telephone calls in the middle and/or at the end of each quarter. |
| **Automatic reminders or notifications via the digital platform**  (11,13,20,25) | V@Si platform offers a notification and reminder system by e-mail or via the platform when there are questionnaires to fill in, as well as scheduled physical activity sessions, workshops or telephone calls. |
| **Social support or experience sharing via the connected platform**  (13,21,26) | Practice of physical activity in a group, together with the possibility of contacting other program participants via the platform chat open during sessions, gives patients the opportunity to share their experience and provides a degree of social support from other patients. |

In addition, our literature review identified five characteristics of interventions described as effective in terms of increasing physical activity levels. These are: ease of use of the digital platform (24,33), feedback (4,21), the possibility of exchanges between participants from the connected platform (21,33), telephone counselling sessions to complement the digital platform (4) and "tailored" interventions (34). The TREVISE program combines all five of these features.

Finally, during our literature review, we found no programs based on videoconferencing that offered professional and collective support during physical activity sessions. In the programs found in the literature, physical activity sessions are often carried out autonomously and described either via written descriptions (texts and images) or via videos filmed beforehand: no interaction is possible during the sessions. In these programs, the use of videoconferencing enables patients to interact not only with the adapted physical activity teacher, but also with other patients, making the program more interactive and reinforcing certain conditions of adherence described in the results of the literature review, such as the link with healthcare professionals (4,9,14,16) or the sharing of experience (13,21,26). TREVISE and PACTIMe are therefore original and innovative programs that offer real added value in terms of interactivity and communication compared with existing programs.

So, based on this analysis, we can assume that the TREVISE and PACTIMe programs may be able to generate strong patient adherence and acceptability, as well as significant results in terms of increased levels of physical activity.

References

1. Tricco AC, Lillie E, Zarin W, O’Brien KK, Colquhoun H, Levac D, et al. PRISMA Extension for Scoping Reviews (PRISMA-ScR): Checklist and Explanation. Ann Intern Med. 2 oct 2018;169(7):467‑73.

2. Golsteijn RHJ, Bolman C, Peels DA, Volders E, de Vries H, Lechner L. A Web-Based and Print-Based Computer-Tailored Physical Activity Intervention for Prostate and Colorectal Cancer Survivors: A Comparison of User Characteristics and Intervention Use. J Med Internet Res. 23 2017;19(8):e298.

3. Kanera IM, Willems RA, Bolman CAW, Mesters I, Verboon P, Lechner L. Long-term effects of a web-based cancer aftercare intervention on moderate physical activity and vegetable consumption among early cancer survivors: a randomized controlled trial. Int J Behav Nutr Phys Act. 10 2017;14(1):19.

4. Lee H, Uhm KE, Cheong IY, Yoo JS, Chung SH, Park YH, et al. Patient Satisfaction with Mobile Health (mHealth) Application for Exercise Intervention in Breast Cancer Survivors. J Med Syst. 6 nov 2018;42(12):254.

5. Ainsworth MC, Pekmezi D, Bowles H, Ehlers D, McAuley E, Courneya KS, et al. Acceptability of a Mobile Phone App for Measuring Time Use in Breast Cancer Survivors (Life in a Day): Mixed-Methods Study. JMIR Cancer. 14 mai 2018;4(1):e9.

6. Martin EC, Basen-Engquist K, Cox MG, Lyons EJ, Carmack CL, Blalock JA, et al. Interest in Health Behavior Intervention Delivery Modalities Among Cancer Survivors: A Cross-Sectional Study. JMIR Cancer. 11 févr 2016;2(1):e1.

7. Forbes CC, Blanchard CM, Mummery WK, Courneya KS. Feasibility and Preliminary Efficacy of an Online Intervention to Increase Physical Activity in Nova Scotian Cancer Survivors: A Randomized Controlled Trial. JMIR Cancer. 23 nov 2015;1(2):e12.

8. Timmerman JG, Dekker-van Weering MGH, Stuiver MM, Groen WG, Wouters MWJM, Tönis TM, et al. Ambulant monitoring and web-accessible home-based exercise program during outpatient follow-up for resected lung cancer survivors: actual use and feasibility in clinical practice. J Cancer Surviv Res Pract. déc 2017;11(6):720‑31.

9. De Cocker K, Charlier C, Van Hoof E, Pauwels E, Lechner L, Bourgois J, et al. Development and usability of a computer-tailored pedometer-based physical activity advice for breast cancer survivors. Eur J Cancer Care (Engl). sept 2015;24(5):673‑82.

10. Gehring K, Kloek CJ, Aaronson NK, Janssen KW, Jones LW, Sitskoorn MM, et al. Feasibility of a home-based exercise intervention with remote guidance for patients with stable grade II and III gliomas: a pilot randomized controlled trial. Clin Rehabil. mars 2018;32(3):352‑66.

11. Schwartz AL, Biddle-Newberry M, de Heer HD. Randomized trial of exercise and an online recovery tool to improve rehabilitation outcomes of cancer survivors. Phys Sportsmed. mai 2015;43(2):143‑9.

12. Hong YA, Goldberg D, Ory MG, Towne SD, Forjuoh SN, Kellstedt D, et al. Efficacy of a Mobile-Enabled Web App (iCanFit) in Promoting Physical Activity Among Older Cancer Survivors: A Pilot Study. JMIR Cancer. 26 juin 2015;1(1):e7.

13. Robertson MC, Tsai E, Lyons EJ, Srinivasan S, Swartz MC, Baum ML, et al. Mobile Health Physical Activity Intervention Preferences in Cancer Survivors: A Qualitative Study. JMIR MHealth UHealth. 24 janv 2017;5(1):e3.

14. Marthick M, Dhillon HM, Alison JA, Cheema BS, Shaw T. An Interactive Web Portal for Tracking Oncology Patient Physical Activity and Symptoms: Prospective Cohort Study. JMIR Cancer. 21 déc 2018;4(2):e11978.

15. Paxton RJ, Hajek R, Newcomb P, Dobhal M, Borra S, Taylor WC, et al. A Lifestyle Intervention via Email in Minority Breast Cancer Survivors: Randomized Parallel-Group Feasibility Study. JMIR Cancer. 21 sept 2017;3(2):e13.

16. M Quintiliani L, Mann DM, Puputti M, Quinn E, Bowen DJ. Pilot and Feasibility Test of a Mobile Health-Supported Behavioral Counseling Intervention for Weight Management Among Breast Cancer Survivors. JMIR Cancer. 9 mai 2016;2(1):e4.

17. Soh JY, Cha WC, Chang DK, Hwang JH, Kim K, Rha M, et al. Development and Validation of a Multidisciplinary Mobile Care System for Patients With Advanced Gastrointestinal Cancer: Interventional Observation Study. JMIR MHealth UHealth. 7 mai 2018;6(5):e115.

18. Kanera IM, Willems RA, Bolman CAW, Mesters I, Zambon V, Gijsen BC, et al. Use and Appreciation of a Tailored Self-Management eHealth Intervention for Early Cancer Survivors: Process Evaluation of a Randomized Controlled Trial. J Med Internet Res. 23 2016;18(8):e229.

19. Lee MK, Yun YH, Park HA, Lee ES, Jung KH, Noh DY. A Web-based self-management exercise and diet intervention for breast cancer survivors: pilot randomized controlled trial. Int J Nurs Stud. déc 2014;51(12):1557‑67.

20. Lloyd GR, Oza S, Kozey-Keadle S, Pellegrini CA, Conroy DE, Penedo FJ, et al. Breast cancer survivors’ beliefs and preferences regarding technology-supported sedentary behavior reduction interventions. AIMS Public Health. 2016;3(3):592‑614.

21. Pope Z, Lee JE, Zeng N, Lee HY, Gao Z. Feasibility of smartphone application and social media intervention on breast cancer survivors’ health outcomes. Transl Behav Med. 01 2019;9(1):11‑22.

22. Galiano-Castillo N, Cantarero-Villanueva I, Fernández-Lao C, Ariza-García A, Díaz-Rodríguez L, Del-Moral-Ávila R, et al. Telehealth system: A randomized controlled trial evaluating the impact of an internet-based exercise intervention on quality of life, pain, muscle strength, and fatigue in breast cancer survivors. Cancer. 15 oct 2016;122(20):3166‑74.

23. Kanera IM, Bolman CAW, Willems RA, Mesters I, Lechner L. Lifestyle-related effects of the web-based Kanker Nazorg Wijzer (Cancer Aftercare Guide) intervention for cancer survivors: a randomized controlled trial. J Cancer Surviv Res Pract. 2016;10(5):883‑97.

24. Short CE, Finlay A, Sanders I, Maher C. Development and pilot evaluation of a clinic-based mHealth app referral service to support adult cancer survivors increase their participation in physical activity using publicly available mobile apps. BMC Health Serv Res. 16 2018;18(1):27.

25. Van Blarigan EL, Chan H, Van Loon K, Kenfield SA, Chan JM, Mitchell E, et al. Self-monitoring and reminder text messages to increase physical activity in colorectal cancer survivors (Smart Pace): a pilot randomized controlled trial. BMC Cancer. 11 mars 2019;19(1):218.

26. Lloyd GR, Hoffman SA, Welch WA, Blanch-Hartigan D, Gavin KL, Cottrell A, et al. Breast cancer survivors’ preferences for social support features in technology-supported physical activity interventions: findings from a mixed methods evaluation. Transl Behav Med. 16 nov 2018;

27. Lynch SM, Stricker CT, Brown JC, Berardi JM, Vaughn D, Domchek S, et al. Evaluation of a web-based weight loss intervention in overweight cancer survivors aged 50 years and younger. Obes Sci Pract. 2017;3(1):83‑94.

28. Baima J, Reynolds SG, Edmiston K, Larkin A, Ward BM, O’Connor A. Teaching of Independent Exercises for Prehabilitation in Breast Cancer. J Cancer Educ Off J Am Assoc Cancer Educ. juin 2017;32(2):252‑6.

29. Park S, Kim JY, Lee JC, Kim HR, Song S, Kwon H, et al. Mobile Phone App-Based Pulmonary Rehabilitation for Chemotherapy-Treated Patients With Advanced Lung Cancer: Pilot Study. JMIR MHealth UHealth. 4 févr 2019;7(2):e11094.

30. Kenfield SA, Van Blarigan EL, Ameli N, Lavaki E, Cedars B, Paciorek AT, et al. Feasibility, Acceptability, and Behavioral Outcomes from a Technology-enhanced Behavioral Change Intervention (Prostate 8): A Pilot Randomized Controlled Trial in Men with Prostate Cancer. Eur Urol. juin 2019;75(6):950‑8.

31. Pfirrmann D, Haller N, Huber Y, Jung P, Lieb K, Gockel I, et al. Applicability of a Web-Based, Individualized Exercise Intervention in Patients With Liver Disease, Cystic Fibrosis, Esophageal Cancer, and Psychiatric Disorders: Process Evaluation of 4 Ongoing Clinical Trials. JMIR Res Protoc. 22 mai 2018;7(5):e106.

32. Forbes CC, Blanchard CM, Mummery WK, Courneya KS. A pilot study on the motivational effects of an internet-delivered physical activity behaviour change programme in Nova Scotian cancer survivors. Psychol Health. 2017;32(2):234‑52.

33. Frensham LJ, Zarnowiecki DM, Parfitt G, King S, Dollman J. The experiences of participants in an innovative online resource designed to increase regular walking among rural cancer survivors: a qualitative pilot feasibility study. Support Care Cancer Off J Multinatl Assoc Support Care Cancer. juill 2014;22(7):1923‑9.

34. Frensham LJ, Parfitt G, Dollman J. Predicting Engagement With Online Walking Promotion Among Metropolitan and Rural Cancer Survivors. Cancer Nurs. 12 oct 2018;
