# Supplementary material for: Conditions for adherence to videoconference-based programs promoting adapted physical activity in cancer patients: a realist evaluation
Source: Implement Sci. 2024 Jan 29;19:6. doi: 10.1186/s13012-024-01338-y (PMC10823602; doi:10.1186/s13012-024-01338-y)
Supplement: Supplementary file 4 — Additional file 4: Appendix 4: Table S2. Interview grid. [file 13012_2024_1338_MOESM4_ESM.docx]

**APPENDIX 4**

**Table 2: Interview grid**

| - - Question 1: What prompted people to enter the program? | | |
| --- | --- | --- |
| - - Question 2: What prompted people to continue the program? | | |
| Follow-up question on the role of peers, relatives and professionals based on the results of the literature review. | | |
| **Factors related to relatives** | | |
| - Family members have a positive attitude towards physical activity and the distance program. - Relatives are physically active - Relatives are or were physically active with the patient. - Family members advise the patient to engage in physical activity for its benefits - Family members help the patient participate in the program remotely - - Patient and family have discussed the program (before and/or during the program)   - Participation of relatives in culinary workshops (optional, open to relatives)  - Negative influence of relatives  - Tips from relatives for resting, sitting or lying down  - Relatives disagree with any physical mobilization (fear of additional fatigue, injuries...)  - Family members give conflicting messages to the oncologist about physical activity and/or the distance program | **Before starting the remote program**   - Positive or negative feedback on the remote program - Perspective of getting out of isolation by finding other patients (motivational lever) | **During the remote program**   - Ripple effect associated with group dynamics - Breaking out of isolation - To have the feeling of being understood, not judged by others in the same situation - -Reduction of the fear of appearing weakened, possibly physically modified (hair loss, weight loss, etc.) - Social learning - Possible exchanges by messenger - Organization of external meetings (at the initiative of the participants) - Emergence of criticism from other participants during the program |
