# Supplementary material for: Conditions for adherence to videoconference-based programs promoting adapted physical activity in cancer patients: a realist evaluation
Source: Implement Sci. 2024 Jan 29;19:6. doi: 10.1186/s13012-024-01338-y (PMC10823602; doi:10.1186/s13012-024-01338-y)
Supplement: Supplementary file 5 — Additional file 5: Appendix 5: Table S3. Factors related to oncologists likely to influence conditions of patients’ adherence. Table S4. Factors related to the patients’ social environment likely to influence their conditions of adherence. [file 13012_2024_1338_MOESM5_ESM.docx]

**APPENDIX 5**

**Table 3: Factors related to oncologists likely to influence conditions of patients’ adherence**

| **Lifestyle** | **Knowledge and attitude** | **Perceived skills** | **Conditions of practice** | **Interactions between oncologists and patients** | |
| --- | --- | --- | --- | --- | --- |
| [LS1] Personal physical activity practice | [KA1] Knowledge about adapted physical activity in oncology (benefits, national and international guidelines, good clinical practice for prescription)  [KA2] Knowledge about the territorial resources providing adapted physical activity to refer patients  [KA3] Knowledge of the remote program (content and expected benefits)  [KA4] Consider that adapted physical activity is compatible with the treatment phase  [KA5] Consider that adapted physical activity is compatible with metastatic cancers  [KA6] Willingness to prescribe adapted physical activity  [KA7] Have a positive view of the remote program  [KA8] Willingness to communicate about physical activity and/or the remote program with patients  [KA9] Willingness to spend time during consultations to discuss adapted physical activity | [PS1] Ease of communicating about adapted physical activity with patients  [PS2] Sense of competence in the prescription of adapted physical activity | [CP1] Opportunity to spend time during consultations to discuss adapted physical activity  [CP2] Possibility and regularity of contact with the managers of the remote program  [CP3] Have received positive feedback from patients about the remote program  [CE4] Have received feedback from the managers of the program about the participation of their patients (referred or not by them) | ***Before starting the remote program***  [IOPB1] The patient has received advice from the oncologist regarding the practice of physical activity  [IOPB2] The patient has received an information document on the benefits of physical activity in oncology (leaflet, flyer)  [IOPB3] The patient has been specifically referred to the remote program  [IOPB4] The patient has received an information document on the remote program  [IOPB5] The patient has received positive feedback from the oncologist about the remote program  [IOPB6] The patient has been examined by the oncologist for his/her participation to the remote program  [IOPB7] The oncologist has provided the medical certificate allowing the practice of physical activity in the context of the remote program | ***During the remote program***  [IOPD1] The patient has been able to talk about his experience of the program during the follow-up consultations  [IOPD2] The patient has been able to give feedback to the oncologist about his or her progress during the program (possibly by giving the oncologist access to his or her personal data), either directly during consultations or by email (via the program platform - intranet messaging, or outside - professional messaging) |

**Table 4: Factors related to the patients’ social environment likely to influence their conditions of adherence**

| **Factors related to relatives** | **Factors related to peers** | |
| --- | --- | --- |
| [REL1] Relatives have a positive attitude towards physical activity and the remote program  [REL2] Relatives are physically active  [REL3] Relatives practice or practiced physical activity with the patient  [REL4] Relatives advise the patient to practice physical activity for its benefits  [REL5] Relatives support the patient to participate in the remote program  [REL6] The patient and his/her relatives have discussed the program (before and/or during the program)  [REL7] Participation of relatives in culinary workshops (optional in TREVISE, open to relatives)  [REL8] Negative influence of relatives  [REL9] Advice from relatives to rest, sit or lie down  [REL10] Relatives disagree with any physical mobilization (fear of additional fatigue, injuries...)  [REL11] Relatives deliver conflicting messages to those from the oncologist about physical activity and/or the remote program | ***Before starting the remote program***  [PEEB1] Positive or negative feedback on the remote program  [PEEB2] Perspective of breaking out of isolation by finding other patients (motivational lever) | ***During the remote program***  [PEED1] Spillover effect associated with group dynamics  [PEED2] Breaking out of isolation  [PEED3] Having the feeling of being understood, non-judged by others who are experiencing the same situation  [PEED4] Reduced fear of appearing weakened, possibly physically modified (hair loss, weight loss, etc.)  [PEED5] Social learning (« vicarious learning »)  [PEED6] Possible exchanges via messaging  [PEED7] Organisation of external meetings (initiative from the participants)  [PEED8] Emergence of criticism during the program from other participants |
