# Supplementary material for: Conditions for adherence to videoconference-based programs promoting adapted physical activity in cancer patients: a realist evaluation
Source: Implement Sci. 2024 Jan 29;19:6. doi: 10.1186/s13012-024-01338-y (PMC10823602; doi:10.1186/s13012-024-01338-y)
Supplement: Supplementary file 6 — Additional file 6: Appendix 6: Table S5. Quotes from participants to illustrate each CMO configuration. [file 13012_2024_1338_MOESM6_ESM.docx]

**APPENDIX 6**

**Table 5: Quotes from participants to illustrate each CMO configuration**

| 1. **An accessible resource and a supportive environment for adapted physical activity**   “The fact that I had to make long trips limited my physical activity enormously, because I had difficulty motivating myself, you know, sometimes there are days when you don't feel like it, and the fact that it's in video, you remove a lot of constraints, you don't really need to prepare beforehand, you only need five minutes before putting on your gym clothes and turning on your computer. So there are so few constraints, you don't have much of an excuse not to motivate yourself, or rather to say "I'm not doing it, I'm not going". So I think I've been more consistent than on programs that require me to go out. For me, it's a good fit for people like me who have an illness, who like me can be tired, lack motivation, so you have days with, days without, and by greatly reducing the constraints associated with it, I think it makes it easier to stick to the program.”  “I tell myself that it's not a big deal, I just have to get dressed and turn on my computer, I say to myself "Go anyway, even if you're not in the best shape of your life, it doesn't matter". If I had to get dressed, go out, take public transport to go there, I wouldn't have done it, there are several times when having this ease of access is already a very big positive point”  “And I could never get seats like nowhere else. For once, there I had a place. A place that I can do and in a different context than going to a hospital. Because even the people who go to the hospital to do their courses, they go back to the medical environment to do something more fun. Well, they come back to this environment where we go to consult, where we are a number, where we have a number. And then, you are at home. You don't have to run all the way to the hospital for that class. At the same time, some people miss not seeing each other, I can understand that. But for me, who has difficulty moving around and getting out of my house. This was the solution that allowed me to do something, to move my body, to regain confidence in my body without having to run, to take transportation”  “I had lost everything, I was confined, I was in a monstrous stress. [...] So I clung to it like I would cling to a lifeline. I said to myself that for once I was being offered something I could do, and that might help me. At first I was very reluctant. I said to myself, "Oh, my, my! The computer and me, that's two. This is not going to work. I won't be able to connect. [It's not for me. And then in the first class, I thought, "Wow! I got online and I did a distance learning course. It was like I was an alien coming to Earth. It was miraculous, like a person discovering, I don't know, something new, incredible”  “Sometimes, the Internet will cut off, we receive the Internet badly or, as I said, when we speak, sometimes there are big gaps, there are people who have more or less a good Internet flow, so the conversation is less fluid as if we were speaking indirectly, sounds a little interspersed, a little bit out of sync, so it's weird”  “There, what is good, it is that we are not much by course, therefore it looks at each people. There are four of us on camera, but sometimes we don't manage to put the camera on us, or sometimes we do something on the ground, so we have to lower the camera and we only see my legs [...] But in any case, it's fine, I remain confident, I know that he is watching the video. I'm careful too, but it didn't block me on one side where I wasn't reassured. At first, I was thinking, will he see that I'm not doing the right position so that I don't get hurt or so that I don't... I don't know... make a bad move. In the end, it's true that it goes” |
| --- |
| 2. A setting conducive to sociability despite the distances  “I can't get out of my house anymore. So it was a real solution to move around and see people [...] And then I also saw colleagues like me, as I call them my friends who had the same pathology as me [...] This course gives me a real moment of conviviality in my week, in addition to movement, something I have not done for some time. And then real encounters because all these people are like me”  “We motivate each other. We see that there are some who can do certain movements, others not. So [...] we say to each other: "I can't do it, I'm not alone. I don't feel inferior. We are all on the same scale. And then we all suffer from the same pathology. So it strengthens the bonds to know that we are all in the same boat but that we manage to move a little once a week with a coach who takes us in hand”  “It turns out that there are women who do not have the same cancer as me. I don't know, but I don't know what stage they're at either. I don't know if they are living well, or if they are not living well at this time. We're all different, but when we all get together, we're all the same. We are all equal, whether we are older or younger. I am fifty-five years old, so there are girls who are very old. And there are girls who are much younger too. And that day, we are all united by the same bond, which is our gymnastics class. And it's important”  “The fact of being.... To know that we were with other people who were going through a little bit of the same thing as us. It made us feel good. Sometimes we had people who were really struggling, who couldn't do much. And then we had some people who were in great shape. So that made you want to do it because you could see the person who was having a hard time one moment, and then they weren't doing so well the next. So it was... It reassured us of our way of seeing things”  “I often feel less tired. It sounds strange, but many people say so. To be less tired after than before, it is not the same fatigue. It's a fatigue, more I would say physical, which seems more natural than the fatigue a bit: "I don't feel like doing anything. Sometimes it's hard. Sometimes I go backwards a little bit, but I go because there's the... There were three of us. There were three other people from the beginning. And in the two courses I'm taking, and in the end, I'll say, we got along well together [...] it encourages us to continue, to go because there's goodwill even if we don't really want to. And then we force ourselves a little. We know that if it's a little too hard, we'll do it a little less hard, but we'll do it a little anyway”  “As I said, I used to do a little at home, but you had to motivate yourself to do it. But now we see that everyone is doing it. And when we are a little bit tired, we feel together to do the exercises. And also if we are tired, we understand very well too. It is also benevolent [...] we are all in the same situation, so there are less prejudices and constraints. If we don't succeed, it's not a big deal”  “And it's the fact that it's a small group too. It allows us to talk about each other's lives. We don't necessarily go into details, but it goes a little bit beyond just doing physical activity. We know a little bit about what people are doing.”  "No one-on-one exchanges like that but we have a little bit of humor. We have a verbal exchange. [...] But yes, there is an exchange, a real exchange. And then we have the impression without knowing each other that we understand each other”  “To do it in a group and to motivate ourselves all together, to be able to talk a little bit with the people. What is good is that we keep the same group in general, so we know each other a little bit. We know who is capable of doing what and then we can evolve together”  “Because these are girls who, for me, are up to the task and are listening and are in full empathy [...] well they ask me on a scale of 1 to 10 how tired I am, but apart from that, unless I have something that day, we don't dawdle on the state of health, we don't talk about the illness. We, that's not their role and that's fine actually. Or else it wouldn't be physical sessions, it would be verbal sessions and that's not the point [...] they're very, very good listeners, but they don't dwell on the condition, on the illness”  “I have a very bad connection. I have a very old computer. So I have a very big gap between the image and the sound compared to other people. But people have adapted to me in a way that I am a little bit late in all the movements. That is to say that I start all the movements well after the others, and I stop well after the others for them. Whereas I feel in sync with her when I look at my screen” |
| 1. **Building confidence and securing practices**   “Right from the start, it made me feel much more secure [...] Plus, I arrived right after my surgery. So it really helped me to move. I had been stuck, I was not moving anymore in fact. I was just doing little steps and he showed me how to do exercises because it's true that compared to the exercises I was doing before, I couldn't necessarily do them again, but in an adapted way and, as a result, he showed me the movements, what I could do and that means that I can do sports. So I liked it because, if I was in pain or if I didn't feel it when I was doing the movements, I never had to do it because finally from the beginning he gave me easy movements to do to start and progressively it increased”  "It's adapted sport, so really, it's an important point, in fact, you are with people who have the pathology, who have limitations like you, and who understand that we can't necessarily do everything, and with teachers who are educated on the issue, and who are always ready to adapt the exercises if they pose a problem, who pay much more attention to all that, to the fact that we may or may not have pain [... ...] here, we are really dealing with things that are adapted, that are thought out for people with the same limitations as we have, and we are surrounded by people who have the same limitations, so I think that we will be more comfortable saying that we can't do it, that it's a problem for us...”  *"And we had a private class, we'll say with a coach who was on the other side of the screen and who showed us the movements, who counted with us, who gave us tricks that allowed us to do movements that we couldn't do otherwise in other positions so that we could still move. And then always with lots of motivation, lots of little encouragements, very human things, we'll say "*  "And even if we are not on the same physical level, the coaches make exercises adapted to each one. If we want to go further, they will give us a more difficult exercise and then the person for example who is a little bit tired or who can't do this movement, they will give him another movement while we do the exercise. So everyone can also do their exercise at different levels. So even if the groups are not homogeneous, it doesn't matter because everyone will have their own exercise so we can all evolve at our own pace but still do it together”  "We work the whole body, including the head. And so, you feel... During this class, you don't necessarily feel patient. You feel like an actress, like a movie actress doing a role. And as a result, you are no longer in pain, or in listening to your body, you are in what you can do to get better. And to think about something else. A bit like a movie actor who will say: "I am now taking the role of a person, so I am no longer me, I am another". Well, it's a little bit like that. And the fact that we are in videoconference, it is also easy to think of the actor who is behind a screen. As a result, we are the ones doing the course. We have the impression of being someone else during this time. At least I feel like that. I'm not an actress, I'm not anything, but during that hour, I'm not the patient anymore. I am a girl among other girls who was trying to think about other things, and to feel good in our body and to move things that don't move anymore, and that hurt when we move. And that are even traumatic sometimes because we don't dare to move certain parts because we don't feel good in those parts, we don't feel good in that part of the body. But we do it without thinking about it because we work everything. We work as well the places like the ankles which do not hurt us necessarily, and then it is a whole. We are considered as a whole body and not as a tumor. A body and a head in addition, we don't make a distinction in this course. In any case, for me, it's a plus, it's my valve” |
| **4. A combination of several motivational levers in favor of continuous effort and progress**  "They were small groups since there were usually, I'll say four maximum, plus the teacher. And it was more adapted to each one in fact. Probably also because there were fewer people at a time [...] if you have a sore knee, for example, which was my case, you point it out. We adapt the exercises, but we do it all the time. And so, although from the beginning, I finished... I continued and finished oral chemotherapy so every day. Plus I also had radiation therapy, I didn't stop. I think I must have missed a session."  "When someone takes my hand and tells me that on such and such a day, at such and such a time, you're going to get off your butt, and you're going to meet people, even if it's by phone, you're going to talk, you're going to talk, they ask you, so today what did you do, what's up. Because otherwise I tend to... I had a period where I didn't see anyone and I felt alone in the world. Except for the shelves, that was all there was. And at home, I mean, I'm a widow, I just moved out.”  *“So it helps me live. It allows me to see that I can still do my movements despite the pain. [...] Physically, it's rewarding and I tell myself that I'm not ruined. And then psychologically, it's a great support because it's a little daily appointment, well not daily but [...] weekly. It's a bit like having an appointment with a girlfriend to chat. Except that we don't chat, we do sports”*  *"And I always look forward to these meetings with great pleasure. That's it, really. And after these sessions, if sometimes my spirits are a bit low, I find a bit of lightness afterwards. Because my illness is not over. Alas, but it helps me to stand up"*  *"The sessions were good, it depended on the session, but it was pretty fun. It wasn't like at the gym. We play little games, we do little relays, even if we're on video, but it works for us"*  "I think there's a real, shall we say, connection between each person's need and the response to that need [...] I was in another session where it was a little more playful as well, so I appreciate the, shall we say, variety, invention or innovation that people put into it. [...] I think that overall in the last year, I've learned a lot about the body and how to hold yourself and how it can affect your health. Even though I was pretty athletic before. In fact "  *"And the interesting thing is that we are all in the process of overcoming and being resilient because we are together"* |
| **5. Regularity of sessions, progressiveness in the effort and evaluation of progress as a basis for the adoption of a regular physical practice**  "There is a lot of seriousness on the part of the teachers, because right on time, we can be sure that the teacher is there, she is not there two minutes later, she is there [...] we feel that it is settled [...] and it is always the same ritual, that is also good, we are made to evaluate the pain, fatigue and aches and pains, and then we compare at the end of the session, there is the stretching, it is very ritualized I find"  "What's also good is that the coaches are very dynamic and that motivates us to move, to do exercises. And they also listen to us. Anyway, at the very beginning, they ask us how we are doing and at the end too. If, for example, we can't move an arm, we say so at the beginning, during the session we also say if it's not going well, and at the end they also see with us if it was at the level of intensity and if there were exercises that didn't go well..."  "But we had the impression that this young man knew everything about our pathology as if he was living it too. He is really within our reach in every gesture. For example, at the beginning of the session, he asks us how we feel about our fatigue and pain, and we give him a score from one to five. And at the end of the session, he asks us again a note from one to five for the pains. And he explains why. He tells us, "Yes, you have made an effort. So you have more pain at that time. And next time, don't pull on it. So that's it. And you have to listen to your body more. So he is really listening, and then he always asks at the end of the session, if the session was not too hard, in our expectations, physically not too breathless. He is really as if he was next to us and at the end of the session, he took us one after the other to come and tell us: "Well, what was positive? What was negative? And what do you feel afterwards?" [...] Here, he listens to our sensations, our pains, our mind, everything. And he never puts us in difficulty"  *"I find it very helpful because I have a hard time coaching myself and they are actually real coaches. You see, because they don't stop at the session either, they tell me "if you can do this, according to your possibilities. If it's possible you do it, if it's not possible you don't do it, but why don't you try a little walk for half an hour afterwards"*  *"This chance is to have recovered a little mobility because my body was completely atrophied. So it allowed me to do small movements again because, I tell you, I don't run, but to regain mobility, which I couldn't do on my own, even though we all know that the Internet, etc., offers gym sessions, etc., but I couldn't do it on my own”*  "The positive thing is to be able to feel a progression, that's also positive [...] that is to say that after a while you feel that you can do the exercises better than at the beginning, you can do them more times, I just did, at the end of the six months there is an evaluation to see the progression between before and after, and here, for me, we could clearly see that there was an improvement of the physical condition. So I think it's also motivating, it's a pleasure to realize that, well, it works"  *"I used to do it more for fun and I needed to move, but now I think I've taken it to another level of knowledge of strength training and awareness of the impact of exercise on the body actually”*  *"I was able to evolve well, that's clear. Afterwards, I did some pretty advanced exercises, but, likewise, I find that it doesn't feel like a gym, but it's always well done. And so, I continue to finish the program because it's good"* |
