# Supplementary material for: Conditions for adherence to videoconference-based programs promoting adapted physical activity in cancer patients: a realist evaluation
Source: Implement Sci. 2024 Jan 29;19:6. doi: 10.1186/s13012-024-01338-y (PMC10823602; doi:10.1186/s13012-024-01338-y)
Supplement: Supplementary file 7 — Additional file 7: Appendix 7: Table S6. Main participants’ demographic and medical characteristics. [file 13012_2024_1338_MOESM7_ESM.docx]

**APPENDIX 7**

| **Information categories** | **N** |
| --- | --- |
| **Mean age, years** [min.-max.] | 51 [31-71] |
| **Gender**  Female  Male | 16  4 |
| **Cancer type**  Gynecological (breast, ovary, uterus)  Cutaneous  Other | 12  2  6 |
| **Stage of the cancer**  In situ  Métastatic | 9  11 |
| **Treatment phase**  In progress  In monitoring | 12  8 |
| **Treatment type**  Immunotherapy  Infusion chemotherapy  Targeted therapy  Radiotherapy  Not applicable | 5  3  3  1  8 |
| **physical activity program**  PACTIMe  TREVISE | 4  16 |

**Table 6: Main participants’ demographic and medical characteristics**
